# Silica Containing Hybrids Loaded with Ibuprofen as Models of Drug Delivery Systems †

**DOI:** 10.3390/ph18101505

**Published:** 2025-10-07

**Authors:** Yoanna Kostova, Pavletta Shestakova, Albena Bachvarova-Nedelcheva

**Affiliations:** 1 Institute of Metal Science, Equipment and Technologies with Hydro- and Aerodynamics Centre “Acad. A. Balevski”, Bulgarian Academy of Sciences, Shipchenski Prohod Str., 67, 1574 Sofia, Bulgaria; 2 Institute of Organic Chemistry with Centre of Phytochemistry, Bulgarian Academy of Sciences, Acad. G. Bonchev Str., bl. 9, 1113 Sofia, Bulgaria; pavletta.shestakova@orgchm.bas.bg; 3 Institute of General and Inorganic Chemistry, Bulgarian Academy of Sciences, Acad. G. Bonchev Str., bl. 11, 1113 Sofia, Bulgaria

**Keywords:** silica–poly(vinylpyrrolidone)–ibuprofen nanohybrids, sol-gel process, in vitro drug release, solid state NMR

## Abstract

**Background/Objectives:** The present work deals with the sol–gel synthesis of hybrid materials based on a silica–polyvinylpyrrolidone (Si-PVP) system. **Methods:** The nanohybrids have been prepared using an acidic catalyst at ambient temperature. Ibuprofen (IBP) was used as a model substance in the obtained model drug systems, while tetraethyl orthosilicate (TEOS) was used as a silica precursor. Poly(vinylpyrrolidone) (PVP) and IBP were introduced into the reaction mixture as solutions in ethanol using two different approaches: (i) a direct introduction of a drug solution into the reaction mixture during sol–gel synthesis, and (ii) a solvent deposition technique. **Results:** XRD data provide evidence that IBP entrapped in the silica–PVP network is in an amorphous state. By SEM it was revealed that in the adsorbate, the IBP particles possess an average particle size of about 20 μm. Based on the obtained IR and UV-Vis spectral results, the existence of hydrogen bonding of IBF with silica and PVP could be suggested. Solid-state NMR analysis allowed the identification of the presence of both crystalline-like and amorphous phases in the hybrid material prepared by the sol–gel method, while it was demonstrated that in the adsorbate, the rigid crystalline dimeric structure of the drug has been preserved. **Conclusions:** The overall analysis of the structural characteristics of the two materials indicated that in the hybrid material obtained by the sol–gel method, the interactions between the amorphous drug, PVP, and the silica matrix are more pronounced as compared to the adsorbate. An improvement of the drug’s aqueous solubility as well of in vitro drug release profile (up to 8 h) was achieved, demonstrating the potential of the developed drug–silica–organic polymer nanohybrid as a promising drug delivery system.

## 1. Introduction

In recent decades, the sol–gel method has been used to facilitate the synthesis of organic–inorganic hybrids. Its main benefits over other techniques are flexibility, adaptability, and low processing pressure and temperature [1]. The hybrid materials’ properties have been demonstrated to work in concert with the beginning materials’ properties at the nanoscale, which qualifies them for use in upcoming technological applications [2]. As a result, they are typically regarded as cutting-edge, modern materials with prospective uses in a wide range of industries, including biology, optics, mechanics, sensors, electronics, and coatings [3]. The nature and the strength of the interactions between the organic and inorganic components strongly influence hybrid characteristics [4].

Silicon alkoxides, such as tetramethoxysilane (Si(OCH_3_)_4_) or tetraethoxysilane (Si(OC_2_H_5_)_4_), are the basis for a broad class of sol–gel materials. These gels’ low density, low dielectric constant, low thermal conductivity, and high specific area make them ideal for a range of applications [5,6,7]. It should be mentioned that the brittle nature of native silica aerogels restricts their use. Nonetheless, a number of techniques for fortifying their structure have already been established, and more are being developed [8,9].

Several publications have reported the first primary results of the sol–gel fabrication of silica hybrids using polyvinylpyrrolidone (PVP) [10,11]. PVP is a preferred molecule among polymers because of its good physiological compatibility, low toxicity, and solubility in organic and watery solutions. It is widely used because of its great qualities, particularly in the sectors of food, medicine, cosmetics, and other areas that are related to human health [12]. Several studies have found that silica–methylmetacrylate—and silica–PVP nanohybrids can be successfully developed by sol–gel processes at mild conditions. The new solid transparent hybrid materials are characterized by the molecular-level dispersion of the organic polymer in the three-dimensional network of silica oxide and could be applied as carriers of drug adsorbates [13,14].

After the 1980s, the sol–gel reaction has been attracting researchers’ interest because it reveals a unique possibility to develop drug systems by introducing a drug solution into the reaction mixture [15,16,17,18]. After the drying, the resulting xerogels contain drug molecules entrapped in the hybrid network. The development of advanced drug delivery systems has garnered significant attention in recent years, particularly for improving the bioavailability and therapeutic performance of poorly water-soluble drugs. Mesoporous silica materials and silica-based hybrids have emerged as versatile platforms for drug encapsulation, owing to their high surface area, tunable pore structure, thermal stability, and chemical inertness [19,20]. Despite a number of challenges, the field’s advancement over the past 10 years shows increasing optimism for novel silica-based drug delivery techniques with significant commercial promise [21]. Generally, silica-based materials are preferable due to their intrinsic biocompatibility, large surface area, high porosity, and ease of functionalization. These features allow for the incorporation of a wide range of drug molecules, especially poorly soluble compounds, and facilitate the modulation of release behavior through tailored material design. Mesoporous silicas such as MCM-41 and SBA-15 have been extensively studied for drug delivery applications, showing promising results in terms of loading capacity and sustained release. However, these systems can suffer from limitations related to weak drug–matrix interactions and rapid desorption in physiological environments [22,23]. To address these limitations, silica–organic hybrid materials—prepared via sol–gel or post-synthetic functionalization routes—have emerged as an attractive class of materials for pharmaceutical applications. These hybrids combine the robustness and porosity of an inorganic silica framework with the tunability of organic functional groups, allowing for improved drug–carrier interactions. The organic component can be selected to interact specifically with target drug molecules, enabling non-covalent stabilization and better control over release kinetics. Additionally, the hybrid structure may offer further control over matrix degradation and surface reactivity in biological media [24].

Ibuprofen (IBP), a nonsteroidal anti-inflammatory drug (NSAID) commonly used for the treatment of pain and inflammation, presents formulation challenges due to its low aqueous solubility and potential for gastrointestinal irritation. The incorporation of ibuprofen into mesostructured silica matrices has been shown to enhance its dissolution profile and mitigate side effects through sustained or targeted delivery. Furthermore, the use of organic–inorganic silica hybrids—materials in which an organic component is covalently integrated into the silica framework—allows for tailored drug–matrix interactions and improved control over drug release dynamics [25].

Using the sol–gel process, the current study aims to develop and explore silica–polymer hybrid materials loaded with ibuprofen as drug delivery model systems. The physicochemical, structural, and morphological characterization of the materials are explored, with special attention paid to the in vitro release behavior and equilibrium solubility of the drug systems. It is investigated how hybrid composition affects the way ibuprofen interacts with the silica matrix in order to provide insight into the design principles that control hybrid drug delivery systems. The novel discoveries will enhance our comprehension of the connections between structure and properties in hybrids.

## 2. Results and Discussion

The materials investigated in this study are denoted as follows: (i) Si-PVP—a hybrid silica–polyvinylpyrrolidone material obtained by sol–gel synthesis; (ii) Si-PVP-IBP (hybrid)—a hybrid material obtained by the direct introduction of a drug solution into the reaction mixture during sol–gel synthesis, and (iii) Si-PVP/IBP (adsorbate), a material obtained by the introduction of a drug into preliminary prepared Si-PVP using the solvent deposition technique.

To evaluate the physico-chemical characteristics and the behavior of the Si-PVP-IBP hybrid as a model drug delivery system, the studies were comparatively carried out with the corresponding pure (Si-PVP) hybrid and the Si-PVP/IBP adsorbate.

The results indicated that PVP was a very suitable organic polymer to develop drug-loaded inorganic–organic hybrids at low processing temperature. The polymer enables molecular level interactions via its N-alkyl substituted carboxylic amide groups with both the silanol groups of the silica structure [26] and the carboxylic OH of IBP. As a result of these interactions during the sol–gel process, solid, transparent, and homogeneous glassy drug hybrid materials were formed.

### 2.1. XRD and SEM Investigations

**Figure 1:**
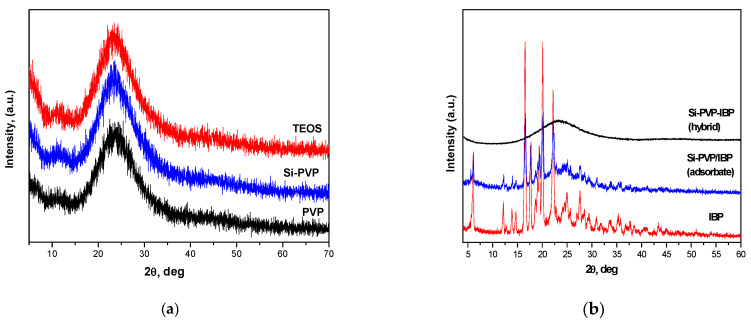
XRD patterns of (**a**) Si-PVP, TEOS, and PVP, and (**b**) Si-PVP-IBP (h), Si-PVP/IBP (a), IBP.

The XRD patterns of all investigated samples are shown in Figure 1a,b. The used precursors (TEOS and PVP) as well as the drug carrier Si-PVP are amorphous (Figure 1a). Figure 1b exhibited the XRD patterns of the model drug systems. As is seen from the figure, the IBP is a crystalline compound, but its incorporation in the investigated model drug systems (Si-PVP-IBP hybrid) stimulates its amorphization (Figure 1b). Therefore, a drug phase transition takes place during the hybrid formation process, and the additional proof of the drugs’ amorphous state is the amorphous halo in the X-ray diffractogram of the same model (Figure 1b). It has to pay attention to the XRD pattern of the Si-PVP/IBP (adsorbate), which keeps the main diffraction lines 2θ = (6.11, 16.61, 17.61, 20.16, and 22.34) [27,28] of IBP but the rest became wider with decreased intensities as a result of the physical interaction in the mixture. Thus, a partial amorphization occurred in that sample. The XRD pattern of the hybrid material is completely amorphous (Figure 1b).

The specific surface area (SSA) results utilizing Brunauer–Emmett–Teller (BET) nitrogen adsorption experiments showed that SSA for the samples Si-PVP, Si-PVP-IBP (hybrid), and Si-PVP/IBP (adsorbate) are with low values 2.82, 3.20, and 4.53 m^2^/g, respectively.

Generally, it was found that the presence of PVP in the silica matrix leads to a decreasing specific surface [29]. There is a slight increase in the surface area after the ibuprofen loading. The highest value of 4.53 m^2^/g is obtained for the adsorbate prepared by the solvent-deposition method. This increasement could probably be related to the washing out of the impurities on the hybrid that purify and activate the surface area of the adsorbate. Additionally, the partial surface adsorption of ibuprofen on the hybrid surface may influence the SSA increase. On the other hand, the reason for the low SSA value of the hybrid (3.20 m^2^/g) could be the higher amount of PVP which enhances the formation of a non-porous structure where the polymer fills the available spaces in the silica network.

**Figure 2:**
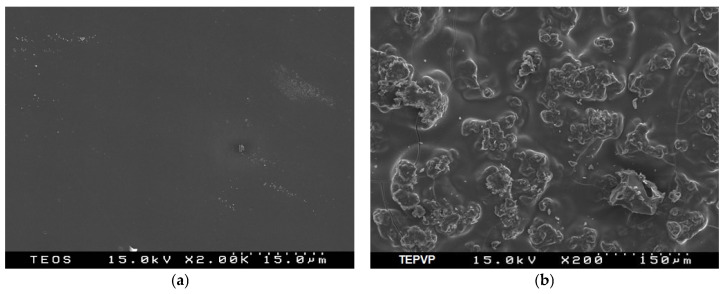

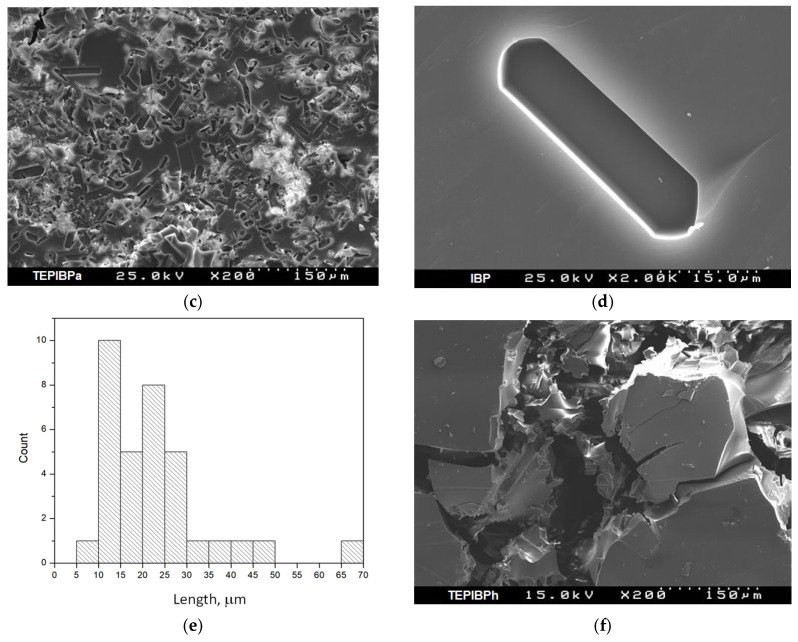
SEM images of pure TEOS (**a**), Si-PVP (**b**), Si-PVP/IBP (adsorbate) (**c**), pure ibuprofen (**d**), particle size distribution of IBP in the adsorbate (**e**), and Si-PVP-IBF (hybrid) (**f**).

Aiming to investigate the particle morphology of the pure carriers and the drug-loaded models, SEM analysis was performed (Figure 2a–e). The SEM images of both TEOS and the Si-PVP hybrid are amorphous (Figure 2a,b) which has been confirmed by the XRD analysis (Figure 1a). It is also worth noting that the particle morphology of the Si-PVP-IBP hybrid is very similar to that of the pure Si-PVP (Figure 2b). No IBP crystals (Figure 2d) can be seen in the SEM picture of Si-PVP-IBF (hybrid) (Figure 2e).

The SEM micrograph of the Si-PVP/IBP (adsorbate) (Figure 2c) shows that its particle morphology is different from that of pure IBP. Moreover, it is clearly shown that the drug crystals are deposited on the surface of the hybrid carrier. Bearing in mind the above mentioned, it could be suggested that IBP molecules crystalize on the surface of the carrier particles, and weak chemosorption occurs, also shown by the XRD data (Figure 1b). Figure 2e exhibited the histogram of the particle size distribution, and it shows that the average particle size of crystalline IBP is about 25 μm.

### 2.2. IR Structural Investigations

**Figure 3:**
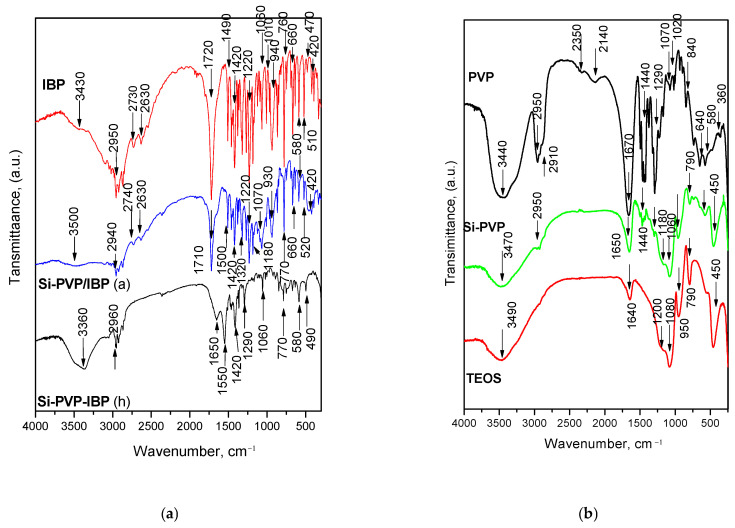
IR spectra of the (**a**) Si-PVP-IBP (h), Si-PVP/IBP (a), IBP, and (**b**) Si-PVP, TEOS, and PVP.

Infrared spectroscopy was used, aiming to obtain additional information on the PVP interaction with the silica surface of the adsorbate Si-PVP/IBP as well as of the IBP incorporation in the hybrid Si-PVP-IBP. All IR spectra are shown in Figure 3a,b. The IR spectra could be divided into two regions—bands below and above 2000 cm^−1^.

The IR spectra of both Si-PVP-IBP models are shown together with the spectra of pure IBP, (Si-PVP) hybrid, and Si-PVP/IBP adsorbate in Figure 3a. As is seen from the figure, several peculiarities have been observed. The typical band of the IBF is centered about 1720 cm^−1^, and it could be assigned to the carbonyl stretching vibrations in hydrogen-bonded dimers [30,31]. It is also presented in the IR spectrum of Si-PVP/IBP adsorbate, but it is shifted to 1650 cm^−1^ in the spectrum of the hybrid material. In the spectrum of the Si-PVP-IBP hybrid, the characteristic stretching C=O vibrations from the PVP amide carbonyl which normally appear as a band at 1670 cm^−1^ are also shifted to lower frequencies with about 20 cm^−1^ and can be observed at about 1650 cm^−1^. A similar phenomenon can be seen in the spectrum of the pure Si-PVP hybrid. This fact can be related to the powerful ability of PVP amide carbonyl to form hydrogen bonds [32,33]. On the other hand, significant changes in the region of the bending C-H vibrations (1500–1420 cm^−1^) which are typical for IBP and PVP, appeared in the spectra of the Si-PVP-IBP drug hybrid model and the Si-PVP hybrid carrier. The sample of pure silica xerogel does not absorb in this region (Figure 3b). The spectrum of the Si-PVP hybrid is similar and consists of the characteristic peaks of the pure silica xerogel (TEOS) (Figure 3a) [34]. However, it is important to outline the changes in the absorption bands which are typical for the pure silica xerogel that were observed in the spectra of the Si-PVP-IBP hybrid and the IBP/hybrid adsorbate. The characteristic band at 950 cm^−1^ in the IR spectrum of TEOS could be assigned to the vibrations of free Si-OH groups on the surface of the amorphous solid that is shifted to 930 cm^−1^ in the IR spectrum of adsorbate. The band at 790 cm^−1^ related to the symmetric ν Si-O-Si bonds is typical for the TEOS and it is presented in the spectra of both model drug systems (Figure 3a) but it is shifted to 780 cm^−1^. It is also seen that the shoulder at 1180 cm^−1^ corresponding to the asymmetric stretching vibrations of Si-O-Si bridging sequences and that vibrations at 450 cm^−1^ resulting from the Si tetrahedron are observed in the Si-PVP-IBP hybrid spectrum, but they are shifted to 1060 and 490 cm^−1^, respectively. This is proof for the interaction between the IBP molecules and the Si-PVP network.

The IR spectra of the Si-PVP-IBP adsorbate model above 2000 cm^−1^ are characterized by two weak bands at 2740 cm^−1^ and 2640 cm^−1^ (Figure 3a). They could be assigned to the O-H stretching and combination vibrations typical for the hydrogen-bonded dimers of the carboxylic acids [35] but they disappeared in the IR spectra of the Si-PVP-IBP hybrid. The obtained results revealed that the IBP participates in interactions with PVP and silica at the expense of dimer formation [36].

### 2.3. UV-Vis Investigations

**Figure 4:**
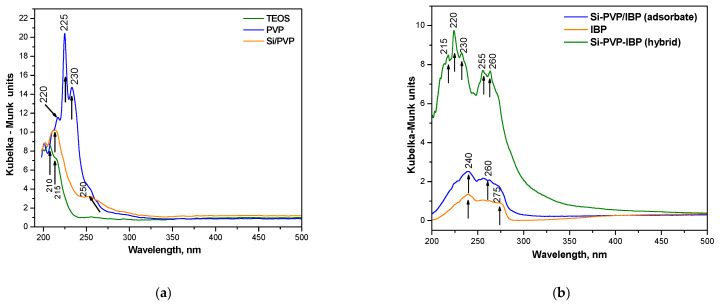
UV-Vis spectra of investigated species: pure TEOS, PVP, and SiO_2_/PVP hybrid (**a**) and the investigated drug systems (**b**).

The UV–Vis spectra in the range from 200 to 500 nm was also used for the determination of the absorption edge and optical band gap of the gels. As is seen from the figure, the absorption peaks were found to be in the range of 210–250 nm. The UV-Vis spectra for pure TEOS, PVP, and the obtained hybrid material are shown in Figure 4a. Pure PVP exhibits a number of absorption peaks at 215, 225, and 235 nm, with the most intense peak occurring at 225 nm. The kind of transitions n → π* that take place between pairs of free charge carriers originating from oxygen atoms and unoccupied states are most likely the cause of the increased absorption of PVP in the UV regions [37]. For UV wavelengths below 230 nm, TEOS demonstrated substantial absorption, which is in line with the findings reported by Xu et al. [38]. Defects in the surface structure of the generated nanoparticles may be the origin of this occurrence, which might lead to the production of an incomplete four-wall network of Si-O-Si that is only characterized by local optical activity [38]. A prominent absorption edge at 220 nm characterizes the spectra of the produced hybrid Si-PVP. Furthermore, it was noted that the composite’s absorption was higher than that of the pure TEOS.

The UV-Vis spectra of pure IBP, Si-PVP-IBP (h), and Si-PVP/IBP (a) are exhibited in Figure 4b. As is seen from the figure, the spectrum of pure IBP showed weak peaks in the range 220–275 nm, which are typical for this compound, but their intensity could vary depending on the concentration [39,40]. The UV-Vis spectrum of Si–PVP/IBP (adsorbate) exhibited higher absorption as compared to the pure IBP along with similar peaks centered at about 240 nm and 260 nm. These findings suggested that ibuprofen is present in the mixture and loosely associated on the surface. The slight shift in the band at 260 nm could be related to the weak interaction with the silica/PVP matrix [41]. Several peculiarities are noticed in the spectrum of the Si–PVP–IBP hybrid. It is obvious that this sample represented the strong absorbance and higher intensity of the following peaks: 215, 220, 230, 255, and 260 nm. This could be related to the interaction between the ibuprofen and the matrix (Si-PVP) as well as the possible amorphization of ibuprofen. The broadened and intense absorbance profile suggests an enhanced dispersion or molecular-level distribution of IBP in the hybrid. Our results correlate well with our data reported by other authors [25,42].

### 2.4. NMR Analysis

Solid-state ^29^Si NMR spectroscopy was applied to obtain an insight into the structural characteristics of the silica matrix in the studied materials. In addition, the ^1^H⟶^13^C cross polarization magic angle spinning NMR spectroscopy (^1^H⟶^13^C CP-MAS) was used to investigate possible interactions between the PVP, drug molecules, and the silica carrier [43,44,45,46].

**Figure 5:**
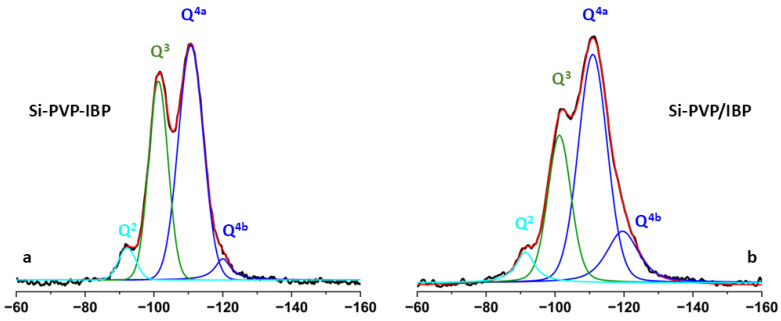
Experimental (black) and simulated (red) direct excitation ^29^Si NMR spectra of (**a**) Si-PVP-IBP and (**b**) Si-PVP/IBP; the individual contributions of the different Si environments obtained after the deconvolution are given with colored lines (Q^4^ blue, Q^3^ green, Q^2^ light blue).

The direct excitation ^29^Si NMR spectra of the Si-PVP-IBP hybrid and the Si-PVP/IBP adsorbate allow quantitative estimation of the different structural environments, (SiO)_n_ Si(OH)_4-n_ species (*n* = 1, 2, 3; e.g Q^n^ species) of the tetrahedrally coordinated Si atoms. Figure 5 presents the experimental and simulated ^29^Si NMR spectra of the hybrid materials as well as the individual contributions of the different Si structural units obtained after the deconvolution of the experimental spectra [47].

The spectra show four resonances that were observed also in the spectrum of the hybrid Si-PVP material, investigated in our previous study [48]; however, with different intensity distributions. The two high field resonances at around −111 (Q^4a^) and −120 ppm (Q^4b^) are characteristic for the Q^4^ structural units [Si(0OH]. The typical Q^4^ chemical shifts in the silicates are at around −110 ÷ −111 ppm, therefore the presence of a second high field resonance indicates that the inclusion of the polymer and IBP resulted in distortion of the bond lengths and valence angles in some Q^4^ building units of the bulk silicate framework. The resonances at around −101.3 ppm and −91.3 ppm are assigned to Q^3^ [Si(1OH) units] and Q^2^ structures [Si(2OH) units], representing defect sites at pore edges and/or at the surface of the silica particles. The relative fractions of the different types of structures calculated by the deconvolution of the ^29^Si spectral patterns are given in Table 1.

The results show that the quantitative distribution of the Q^4^, Q^3^, and Q^2^ structural units varies between the two materials and is different from the one found in the hybrid Si-PVP material from our previous study (Q^4a^:Q^4b^:Q^3^:Q^2a^:Q^2b^ = 40:10:45:4:1) [48]. From the three above-mentioned materials, the adsorbate (Si-PVP/IBP), is characterized by the lowest amount of Q^3^ structures (28%) and the highest quantity of Q^4^ structures (65% in total). Therefore we can conclude that the solvent deposition technique for sample preparation resulted in overall consolidation of the silica network, although the structural heterogeneity within the Q^4^ species (Q^4a^:Q^4b^ = 51:15) was increased as compared to the two other hybrid materials (Q^4a^:Q^4b^ = 51:5 in Si-PVP-IBP, and Q^4a^:Q^4b^ = 40:10 in Si-PVP without IBP). The Si-PVP-IBP hybrid, obtained by sol–gel assisted synthesis, including the three components, has a lower amount of Q^3^ (39%) and a higher amount of Q^4^ units (56% in total) as compared to the Si-PVP material (45% Q^3^ and 50% Q^4^ in total), obtained by the same procedure. In both materials containing IBP, the fraction of the Q^2^ species remained low and almost identical (5–6%).

**Figure 6:**
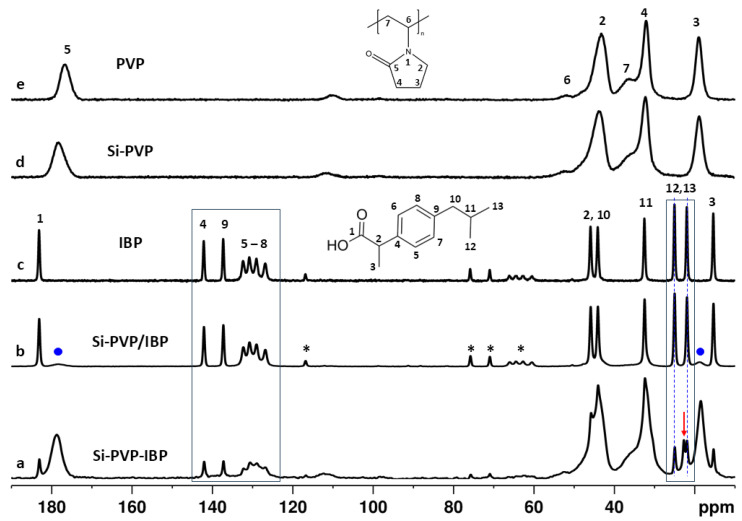
^1^H⟶^13^C CP-MAS spectra of (**a**) Si-PVP-IBP hybrid, (**b**) Si-PVP/IBP adsorbate (the blue dots indicate selected resonances of PVP), (**c**) bulk IBP, (**d**) Si-PVP hybrid, and (**e**) PVP. The chemical structures and corresponding assignments of the ^13^C resonances of IBP and PVP are given for clarity. The areas marked with the two blue rectangles are discussed in the main text. The blue dashed lines indicate the signals of the two methyl carbons from the isopropyl group of crystalline IBP that are identical with those of the bulk IBP, while the red arrow points to the single averaged resonance of the methyl carbons from the isopropyl group in the amorphous state within the silica matrix. The asterisks (*) show the spinning side bands of the aromatic signals resulting from the rotation of the samples at the magic angle.

^1^H⟶^13^C cross polarization magic angle spinning NMR spectroscopy (^1^H⟶^13^C CP-MAS) was used to investigate the possible interactions between the PVP, IBP, and the silicate in the hybrid drug-loaded materials. The ^1^H⟶^13^C CP-MAS spectra of the Si-PVP-IBP hybrid and Si-PVP/IBP adsorbate are presented in Figure 6. The spectra of the pure crystalline IBP, pure PVP, and the hybrid Si-PVP samples are also displayed for comparison.

The spectra of the two drug-loaded samples show relatively narrow resonances originating from IBP as well as the characteristic broad signals of the PVP. The relative intensities of the IBP and PVP resonances are different in the two samples. In the spectrum of the Si-PVP-IBP hybrid, the resonances of the PVP have relatively high intensity and partially overlap the narrow IBP signals in the region from 60 ppm to 10 ppm, while in the spectrum of the Si-PVP/IBP adsorbate, these resonances are hardly visible near the spectral baseline (selected signals of PVP are indicated with dots in Figure 6b). The CP-MAS spectra cannot be interpreted quantitatively since the effectiveness of cross-polarization transfer from ^1^H to the neighboring heteronuclei and hence the magnitude of ^13^C signal enhancement depends on various factors such as the ^13^C→^1^H internuclear distance, the relaxation rates, and local mobility of the structural fragments, which vary from one chemical environment to another. Therefore, the spectra allow only qualitative estimation of the relative amounts of IBP and PVP within each of the studied materials. The spectrum of pure IBP shows distinct narrow resonances for all 13 carbon atoms of the molecule, which is typical for rigid crystalline solid, where the specific molecular packing results in the crystallographic nonequivalence of the different carbons. Previous studies demonstrated that in crystalline ibuprofen the molecules are associated in dimers [49]. The spectral patterns of IBP in the spectra of Si-PVP/IBP adsorbate are identical to those of the bulk IBP, indicating that the drug molecules have crystallized on the surface of the silica particles. The spectrum of the hybrid Si-PVP-IBP material, however, shows slightly different IBP spectral characteristics. This is clearly visible, particularly in the aromatic carbon region from 160 to 140 ppm, (outlined with a rectangle including Figure 6a–c) where the sharp signals of the crystalline-like phase are on top of the broader resonance, pointing towards the presence of an amorphous component. The detection of the amorphous phase in samples with mixed crystalline/amorphous phases is often hampered by the overlap of the broader usually low intensity signals of the amorphous phase by the sharp intense resonances of the crystalline phase. Indeed, the presence of the mobile amorphous phase encapsulated in the silica matrix of the Si-PVP-IBP hybrid cannot be excluded considering the specific sensitivity of the CP experiment to rigid phases. The CP experiment relies on the transfer of magnetization from ^1^H to neighboring ^13^C nuclei that is based on ^1^H-^13^C dipole–dipolar interactions. The molecular mobility averages out these types of interactions, therefore decreasing the efficiency of magnetization transfer resulting in lower enhancement of ^13^C signal intensity for the mobile phases [50]. Additional evidence for the presence of the two phases in the Si-PVP-IBP hybrid could be found in the aliphatic signal region, where there are two distinct resonances for the two methyl carbons of the isopropyl group (at 24.96 and 24.89 ppm), which have different chemical shifts due to their different chemical environments in the crystalline phase. Their chemical shifts are practically identical to those observed in the spectrum of the pure crystalline IBP. However, there is also an additional signal, at 22.64 ppm (indicated with the red arrow in Figure 6a), which is not present in the spectrum of the bulk IBP. This signal originates from the methyl carbons of the isopropyl group of drug molecules in amorphous phase within the silica material. Due to higher molecular mobility and fast reorientation, the chemical environment of the two methyl groups is averaged out, and they become magnetically equivalent, giving one signal in the spectrum [50]. These specific spectral features were not observed in the spectrum of the Si-PVP-IBP adsorbate that is practically identical with the spectrum of the bulk IBP, which rules out the possibility for the presence of the amorphous drug phase incorporated in the silica matrix.

Further analysis of the ^1^H⟶^13^C CP-MAS spectra allowed to elucidate possible interactions between the drug, PVP, and the silica carrier. Comparison of the ^13^C chemical shifts shows that there are slight differences in the chemical shifts in the resonances of drug C=O group in the spectra of the Si-PVP-IBP (183.05 ppm), Si-PVP/IBP (183.09 ppm), and the bulk IBP (183.13 ppm). The carboxylic group resonance is shifted most downfield in the spectrum of the bulk IBP, while it appears most upfield in the spectrum of the Si-PVP-IBP hybrid. These observations are demonstrated in Figure S1 (red dashed line) in Supplementary Materials. Similar trends were observed for some carbons from the aliphatic fragment such as the CH_3_ group close to the carboxylic carbon (C-3) (in Supplementary Materials Figure S2, red dashed line) and four of the aromatic resonances (C-2, C-9 and two of the four CH signals). The chemical shift changes were more pronounced in the spectra of the Si-PVP-IBP hybrid, indicating its involvement in stronger interactions. Chemical shift changes were detected also for the C=O group of PVP (Figure S1, blue dashed line). Our previous study showed that the resonance of the C=O groups in the spectrum of the Si-PVP hybrid is shifted by 1.63 ppm downfield as compared to pure PVP, indicating that the C=O groups of the pyrrolidone polymer fragment are involved in hydrogen bonding interactions with the OH hydrogens of the silanol groups from the Q^3^ and Q^2^ centers of the silica matrix) [48] Further increase in the chemical shift value of the PVP C=O resonance was detected in the drug-loaded samples Si-PVP/IBP (178.26 ppm) and Si-PVP-IBP (178.71 ppm), with a more pronounced low field shift observed of the hybrid Si-PVP-IBP material. The overall analysis of the NMR data implies that carboxylic groups of IBP molecules interact with the PVP and the silica matrix by non-bonding interactions. We suggest that the effect is stronger in the hybrid Si-PVP-IBP material, since the dissociation of the IBP dimers and increased molecular mobility in the amorphous phase within the silica network makes it possible for specific molecular orientations to facilitate these interactions.

**Figure 7:**
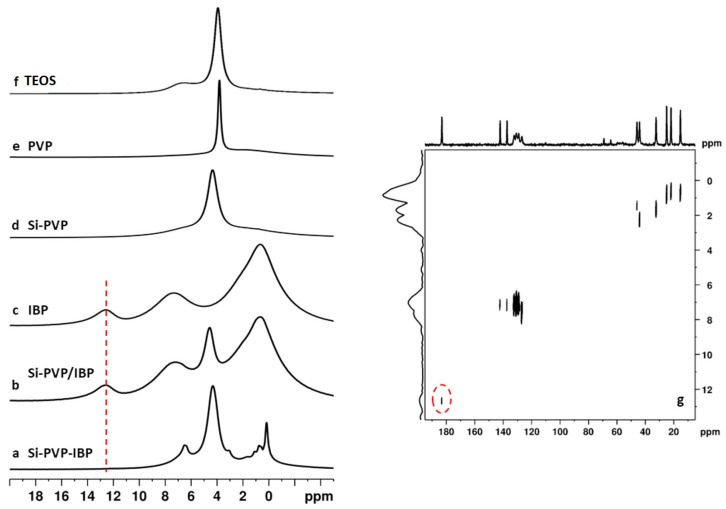
^1^H spectra of (**a**) Si-PVP-IBP hybrid, (**b**) Si-PVP/IBP adsorbate, (**c**) bulk IBP, (**d**) Si-PVP hybrid, (**e**) PVP, and (**f**) silicate (TEOS); (**g**) 2D ^1^H-^13^C HETCOR spectrum of bulk crystalline IBP.

Finally, the analysis of the ^1^H NMR spectra give further evidence about the involvement of the IBP carboxylic functionality in chemical exchange and weak H-bonding interactions. ^1^H solid state spectra of the studied samples are presented in Figure 7a–f. The spectrum of crystalline IBP (Figure 7c) shows three broad spectral patterns centered at around 1.6 ppm, 7 ppm, and 12.8 ppm, corresponding to aliphatic, aromatic, and carboxylic protons. The resonances are broad due to incomplete averaging of the strong homonuclear dipole–dipolar interactions in the rigid crystalline solid. The assignment of the different resonances to the corresponding structural fragments was confirmed by the 2D ^1^H-^13^C HETCOR spectrum of IBP (Figure 7g), where the correlation peaks at short mixing time (100 µs) reflect heteronuclear dipole–dipolar interactions between ^1^H and ^13^C within the given structural fragment. In addition, the ^1^H dimension of the HETCOR spectrum has better resolution as compared to the 1D spectrum allowing us to identify in more detail the ^1^H chemical shifts in IBP. The 2D HETCOR spectrum confirmed that the resonance at 12.8 ppm in the ^1^H spectrum originates from the COOH protons since there is a correlation peak between the signal at 12.8 ppm in the ^1^H dimension and the signal at 183 ppm in the ^13^C dimension of the HETCOR spectrum (outlined with a red dashed circle in Figure 7g). The ^1^H spectrum of the Si-PVP/IBP adsorbate is very similar to those of the bulk IBP with clearly visible resonance of the carboxylic protons at 12.8 ppm. The additional signal at around 4.4 ppm most probably originates from water adsorbed at the silica surface or associated with the PVP, since a resonance with similar chemical shift is observed also in the spectra of the Si-PVP hybrid, the pure PVP, as well as in the pure silica (TEOS) (Figure 7d, e, f, respectively). These observations are in line with the conclusions based on the analysis of the ^13^C spectra, confirming that in the adsorbate, the IBP preserves its rigid crystalline dimeric structure supported by strong H-bonds between the IBP molecules, which prevents its interactions with the PVP and/or the silicate carrier. The ^1^H spectrum of the Si-PVP-IBP hybrid significantly differs from the spectra of both the bulk IBP, as well as the adsorbate Si-PVP/IBP material. The spectrum shows better signal resolution with relatively narrow resonance linewidths and the broad COOH resonance at 12.8 ppm is missing (red dot line in Figure 7a–c). The noticeable narrowing of the IBP resonances supports the conclusion that IBP incorporated in the network of the Si-PVP-IBP hybrid is in an amorphous phase, where its higher mobility averages out the homonuclear dipole–dipolar interactions, resulting in signal narrowing. The absence of the COOH signal could be explained with the possible involvement of the carboxylic protons in chemical exchange with other protons such as the protons from silanol groups (Si-OH) of the silica and/or adsorbed water molecules. In addition, participation in weak H-bonding interactions with the pyrrolidone C=O group of PVP where the carboxylic proton exchanges between the C=O groups from the neighboring polymeric fragments is also a plausible explanation about the absence of the signal at 12.8 ppm in the ^1^H spectrum of Si-PVP-IBP.

### 2.5. Ibuprofen Release Profiles

**Figure 8:**
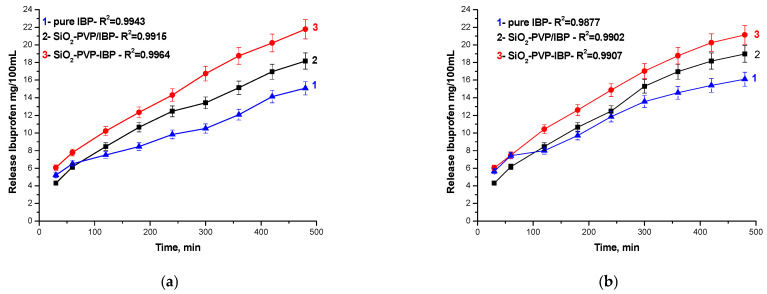
Solubility changes in the IBP release profile up to 8 h of both drug models—in HCl (**a**) and in H_2_O (**b**).

Aiming to investigate the solubility changes in the IBP release, three dissolution media were applied. The obtained nanohybrid drug models were used in order to simulate 0.1 M HCl, water (GI fluids) as well as the phosphate buffer pH 6.8. The release profiles of the adsorbate Si-PVP/IBP and Si-PVP-IBP hybrid drug models are exhibited in Figure 8a,b. The IBP adsorbate profiles are also shown as a reference.

It was determined that, in contrast to the other media, the IBP release in phosphate buffer pH 6.8 (profiles not shown) proceeds quickly and takes around 60 min to complete [51]. As is seen from the figure, the release profiles are very close to that of the pure IBP sample. These phenomena can be related to the buffer alkaline ions, which probably favor the dissolution of the weak acid IBP (Figure 8a,b). Nevertheless, because silica dissolves actively at high pH, the network of samples is destroyed rather quickly. For example, the silica amount dissolved in buffer pH 6.8 from the sample (Si-PVP) pure hybrid at the 8th hour is about 50 times higher than that registered in an acidic medium (Table 2). It is obvious that in 0.1 M HCl and water, both types of IBP nanohybrid models reveal an effective prolonged drug release—up to the 3rd hour, the pure IBP release is ~8 mg/mL while up to the 8th hour, its solubility increases up to ~13 mg/mL (Figure 8a,b). The IBP solubility in the hybrid up to the 3rd hour is ~12 mg/mL, but at the 8th hour, it increases up to 22 mg/mL (Figure 5b).

On the other hand, the profiles of both models in HCl are very similar to those in water. The profiles in water are, however, to some extent higher due to improved dissolution of IBP at pH above its pK_a_ value-4.5 as to the increased silica dissolution at higher pH [52]. It is very probable that the release processes in 0.1 M HCl are influenced by the dissolving poly(vinylpyrrolidone) which improves IBP dissolution because of complex formation with IBP molecules in an acidic medium [53,54]. A similar relationship has been observed with the equilibrium solubility data obtained in an acidic medium at 24 °C (Table 2). In comparison to the solubility of pure IBP, the IBP hybrid solubility is about three times higher than that of the adsorbate.

It is also important to note that the Si-PVP-IBP hybrid’s profile is higher than those of the adsorbate (Si-PVP/IBP) as well as pure IBP (Figure 8a,b), regardless of the nature of the dissolution medium. This phenomenon could be explained by the amorphization of the IBP in the hybrid proved by the XRD analysis (Figure 1b). The amorphization of IBP is a preferable and desirable state for achieving a good solubility of a drug system. This correlates well with results obtained by other authors [55,56].

The changed release profile of the hybrid (Si-PVP-IBP) in comparison to that in the HCl medium can be related to the increased dissolution of silica at higher pH, leading to the network destruction. For example, the silica dissolution of the Si-PVP hybrid in water is about eight times higher than that in hydrochloric acid (Table 2). Moreover, IBP facilitates the dissolution of SiO_2_ in water (0.0047 mg/5ml) because it quits the hybrid network and dissolves more easily at higher pH than in an acidic medium (0.004 mg/5ml). As can be seen from Table 2, the equilibrium solubility of the Si-PVP-IBP model is ~2 times higher than in 0.1M HCl. Based on the observed changes in the release profile, it could be summarized that the hybrid (Si-PVP-IBP) behaves as a modified drug system [55,57].

## 3. Materials and Methods

Alkoxide tetraethyl orthosilicate (TEOS)—Sigma Aldrich Chemie GmbH, Albuch, Germany, Molecular weight: 208.33, reagent grade, 98%; polyvynylpyrrolidon K25, PVP, Polyvidone, Povidone, tested according to Ph. Eur. Linear Formula: (C_6_H_9_NO)_n_, Sigma-Aldrich Chemie GmbH, Density (D) 1,2 g/cm^3^; (±)-2-(4-isobutylphenyl)-propionic acid (IBP), Ibuprofen 50 (Catalytic Process)—BASF Pharma Chemicals, England; and ethanol (C_2_H_5_OH, 99,9%, Aldrich); Hydrochloric acid 36%, SUPRAPUR^®^, Molecular Weight: 36.46, Supelco (Merck, Darmstadt, Germany).

### 3.1. Preparation of Silica-Polyvynynylpyrrolidon (Si-PVP) Hybrid

TEOS, H_2_O, and 0.1 M HCl in the molar ratio 4:1:1 were stirred together on a magnetic stirrer. The appropriate amount of PVP was dissolved in ethanol and added dropwise to the stirred solution. The stirring continued until the mixture clarified. The gelation occurred for ~ 20h at room temperature.

### 3.2. Sol–Gel Assisted Preparation of Silica–Polyvynylpirrolydon–Ibuprofen (Si-PVP-IBP) Hybrid Materials

According to the above-described procedure for the preparation of the Si-PVP hybrid, the IBP amount corresponding to the hybrid ratio Si-PVP/IBP (1:1 *w*/*w*) was added to the mixture after dissolving it in the PVP alcoholic solution.

### 3.3. Characterization of the Prepared Model Systems

Powder X-ray diffraction data were obtained on a Philips PW1730/10 diffractometer using Ni filtered CuKά radiation. The scanning rate for crystallinity was 1.2° 2θ/min. SEM images were obtained on a Hitachi S-4100 (Hitachi Ltd., Tokyo, Japan) microscope at an accelerating voltage of 25.0 kV. FTIR spectra were registered using the KBr pellet technique on the FTIR spectrophotometer—Matson 7000 (Matson, Gloucester, England). The UV-Vis diffused reflectance spectrophotometer “Evolution 300” with a magnesium oxide reflectance standard as the baseline was used for performing the optical absorption spectra of the powdered samples in the wavelength range 200–500 nm. The specific surface areas (BETs) were determined by low-temperature (77.4 K) nitrogen adsorption in the NOVA 1200e (Quantachrome Instruments NOVA 1200e apparatus, Anton Paar GmbH, Graz, Austria) surface area and pore analyzer at relative pressures p/p0 = 0.1–0.3 using the BET equation.

NMR spectra were recorded on a Bruker Avance HD III 600 NMR spectrometer (Bruker Biospin, Ettlingen, Germany) operating at 599.98 MHz proton frequency (150.84 MHz for ^13^C, 119.18 MHz for ^29^Si), using a 4 mm solid state i-CP/MAS dual ^1^H/^31^P-^15^N probehead (Bruker Biospin, Ettlingen, Germany). The samples were loaded in 4 mm zirconia rotors and spun at a magic angle spinning (MAS) rate of 10kHz for both the ^13^C and ^29^Si measurements. The quantitative direct excitation ^29^Si NMR spectra were recorded with one-pulse sequence, 90° pulse length of 4.5 µs, 3 K time domain data points, spectrum width of 70 kHz, 1024 scans, and a relaxation delay of 60 s. An exponential window function was applied (line broadening factor 10) prior to Fourier transformation. ^1^H⟶^13^C cross-polarization MAS (CP MAS) spectra were acquired with the following experimental parameters: 8 K time domain data points, spectrum width of 50 kHz, ^1^H excitation pulse of 3.6 µs, contact time of 2 ms, 2048 scans, and a recycle delay of 5 s. A ^1^H SPINAL-64 decoupling scheme was used during acquisition of the CP experiments. The spectra were processed with an exponential window function (line broadening factor 10) and zero filled to 32 K data points. Octakis(trimethylsiloxy)silsesquioxane powder (Q8M8, δ^29^Si = 11.7 ppm) was used for ^29^Si chemical shift referencing as an external standard. ^13^C chemical shifts were referenced to the carboxyl group peak of α-glycine (δ^13^C = 176.5 ppm).

### 3.4. “Solvent Deposition” Technique for Preparation of a Model Ibuprofen Adsorbate onto Si-PVP Hybrid (Si-PVP/IBP) 1:1 (w/w) Adsorbate

The adsorbate was prepared in a 1:1 (*w*/*w*) ratio. An appropriate amount of (Si–PVP) hybrid material was dispersed with a magnetic stirrer in the IBP solution in ethanol (96% *v*/*v*) placed in a round bottom flask. The suspension was stirred for an additional one hour at room temperature. The solvent was then evaporated at 60 °C in a Rotavapor R-114 (Buechi, Switzerland). Finally, the solid residue was dried overnight over P_2_O_5_.

### 3.5. Equilibrium Solubility

A total of 50 mg Si-PVP-IBP (hybrid) or Si-PVP/IBP 1:1 (w/w) adsorbate, equivalent to 25 mg IBP (excess amount) as well as 25 mg Si-PVP hybrid, were placed in vials with glass stoppers together with 5 mL water and 0.1 M HCl. Subsequently, they were shaken in a thermoregulated water bath at 22 ± 0.5 °C until equilibrium was reached for ~30h. The IBP concentration was determined in a filtered sample by a UV spectrometer at the 264 nm wavelength. A sample of Si-PVP hybrid, treated as above described, was used as a blank (Table 2). SiO_2_ has no absorption at this wavelength.

### 3.6. In Vitro Dissolution Profile of Ibuprofen Models

According to the European Pharmacopoeia, 7th ed, Dissolution apparatus II (paddle method), (Dissolution Tester ERWEKA-DT600, Erweka, Langen, Germany) was used. The amount of the sample was 200 mg. The dissolution medium was 350.0 mL distilled water and 0.1 M HCl; the rotation speed—50 rpm; the temperature 37 ± 0.5 °C. Samples of 5 mL were withdrawn at predetermined time intervals. The IBP concentration was spectrophotometrically determined (UV-spectrophotometer Hewlett-Packard-8452A, Palo Alto, CA, USA) at 264 nm. The same dissolution test was carried out with 100 mg of untreated Si-PVP (hybrid) material. The above-mentioned samples at each time interval were used as a blank (Figure 8).

## 4. Conclusions

Two different techniques (sol–gel process and solvent deposition) have been applied for obtaining drug-loaded silica-poly(vinylpyrrolidone) materials. By XRD, a higher tendency of amorphization of Ibuprofen (IBP) in the sol–gel prepared hybrid sample was established as compared to the adsorbate. The used SEM, IR, and NMR analyses also confirmed this result.

The overall analysis of the NMR data allowed us to identify the formation of a mixed IBP phase composed of crystalline-like and amorphous phases in the hybrid Si-PVP-IBP material. The dissociation of the rigid crystalline dimeric structure and the higher molecular mobility of the IBP molecules entrapped in the silica network facilitated the interactions of the drug with the PVP and silica carrier at the molecular level. On the contrary, in the Si-PVP/IBP adsorbate, the IBP is adsorbed on the surface of the silica particles as a crystalline solid, preserving its dimeric structure supported by strong H-bonds between the IBP molecules, therefore preventing its interactions with the PVP and/or the silicate carrier.

The data obtained indicated that the structural characteristics of the materials under investigation caused differences in the release profile of both samples when compared to pure IBP. Adsorbate and hybrids, two modified drug models, both had higher and longer-lasting drug release properties, which affected IBP’s solubility.

### Limitation of the Study

Although this study demonstrates promising in vitro release behavior of the ibuprofen-loaded silica hybrid system, its in vivo performance has not yet been evaluated. Given the inherently poor water solubility of ibuprofen, our formulation showed improved drug release under laboratory conditions. However, the extent to which this enhancement translates to improved bioavailability and therapeutic efficacy remains to be confirmed in future in vivo studies. The long-term storage stability under controlled environmental conditions will also be a focus in future work.

## Figures and Tables

**Table 1:** Chemical shifts and relative fractions of the different Q^n^ structural units obtained after deconvolution of the direct excitation ^29^Si spectra.

Sample	Q^2^		Q^3^		Q^4a^		Q^4b^	
	ppm	*I*	ppm	*I*	ppm	*I*	ppm	*I*
Si-PVP-IBP	−91.9	5	−101.3	39	−110.8	51	−120.0	5
Si-PVP/IBP	−91.3	6	−101.3	28	−110.9	51	−119.6	15

**Table 2:** Ibuprofen and silica solubility data.

Ibuprofen Solubility Data				Silica Solubility Data	
Equilibrium Solubility of IBP in [mg/5 mL], *n* = 3				Si Amount Dissolved [mg/5 mL] at 8th h, *n* = 2	
Solvent	Pure IBP	IBP Adsorbate	Si-PVP-IBP Hybrid	Si-PVP Hybrid	Silica Xerogel
0.1 M HCl	1.0 ± 0.06	2.22 ± 0.10	3.14 ± 0.10	0.0002	0.0004
water	1.23 ± 0.15	3.17 ± 0.21	5.08 ± 0.21	0.0017	0.0047

## Data Availability

The original contributions presented in this study are included in the article/Supplementary Materials. Further inquiries can be directed to the corresponding authors.

## Supplementary Materials

The following supporting information can be downloaded at https://www.mdpi.com/article/10.3390/ph18101505/s1, Figure S1: Expanded region of the C=O resonances in the ^1^H⟶^13^C CP-MAS spectra of (**a**) Si-PVP-IBP hybrid, (**b**) Si-PVP/IBP adsorbate, (**c**) bulk IBP, (**d**) Si-PVP hybrid, and (**e**) PVP; Figure S2: Expanded region of the aliphatic resonances in the ^1^H⟶^13^C CP-MAS spectra of (**a**) Si-PVP-IBP hybrid, (**b**) Si-PVP/IBP adsorbate, (**c**) bulk IBP, (**d**) Si-PVP hybrid, and (**e**) PVP.
