# Supplementary material for: Silica Containing Hybrids Loaded with Ibuprofen as Models of Drug Delivery Systems
Source: Pharmaceuticals (Basel). 2025 Oct 7;18(10):1505. doi: 10.3390/ph18101505 (PMC12567524; doi:10.3390/ph18101505)
Supplement: Supplementary file 1 [file pharmaceuticals-18-01505-s001.zip › pharmaceuticals-3880032-supplementary.pdf]

pharmaceuticals

MDPI

Supplementary Materials: Silica Containing Hybrids Loaded with Ibuprofen as Models of Drug Delivery Systems

Yoanna Kostova, Pavletta Shestakova and Albena Bachvarova-Nedelcheva

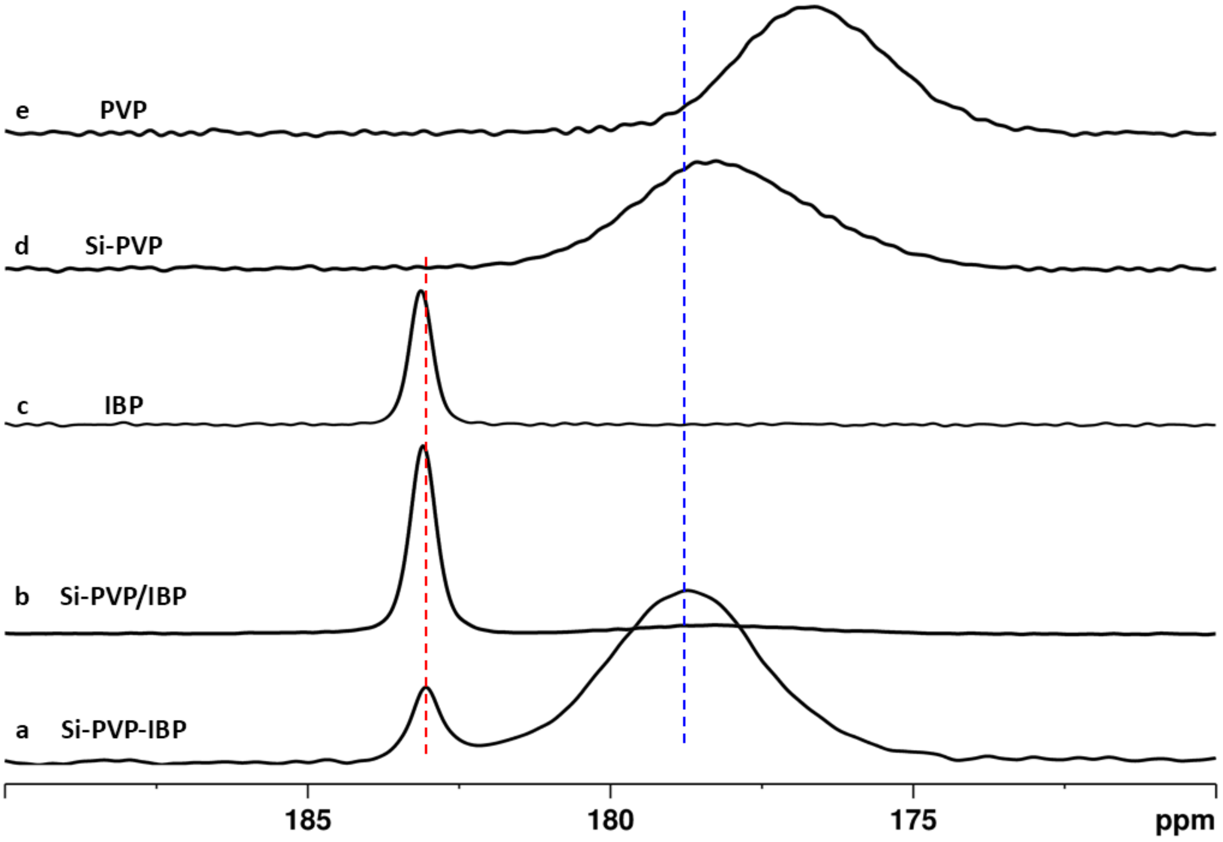

Figure S1. Expanded region of the C=O resonances in the $^{1}H \rightarrow ^{13}C$ CP-MAS spectra of: (**a**) Si-PVP-IBP hybrid, (**b**) Si-PVP/IBP adsorbate, (**c**) bulk IBP, (**d**) Si-PVP hybrid and (**e**) PVP. The dashed lines are guides for the eye for the chemical shift changes of the C=O resonances of IBP (red) and PVP (blue).

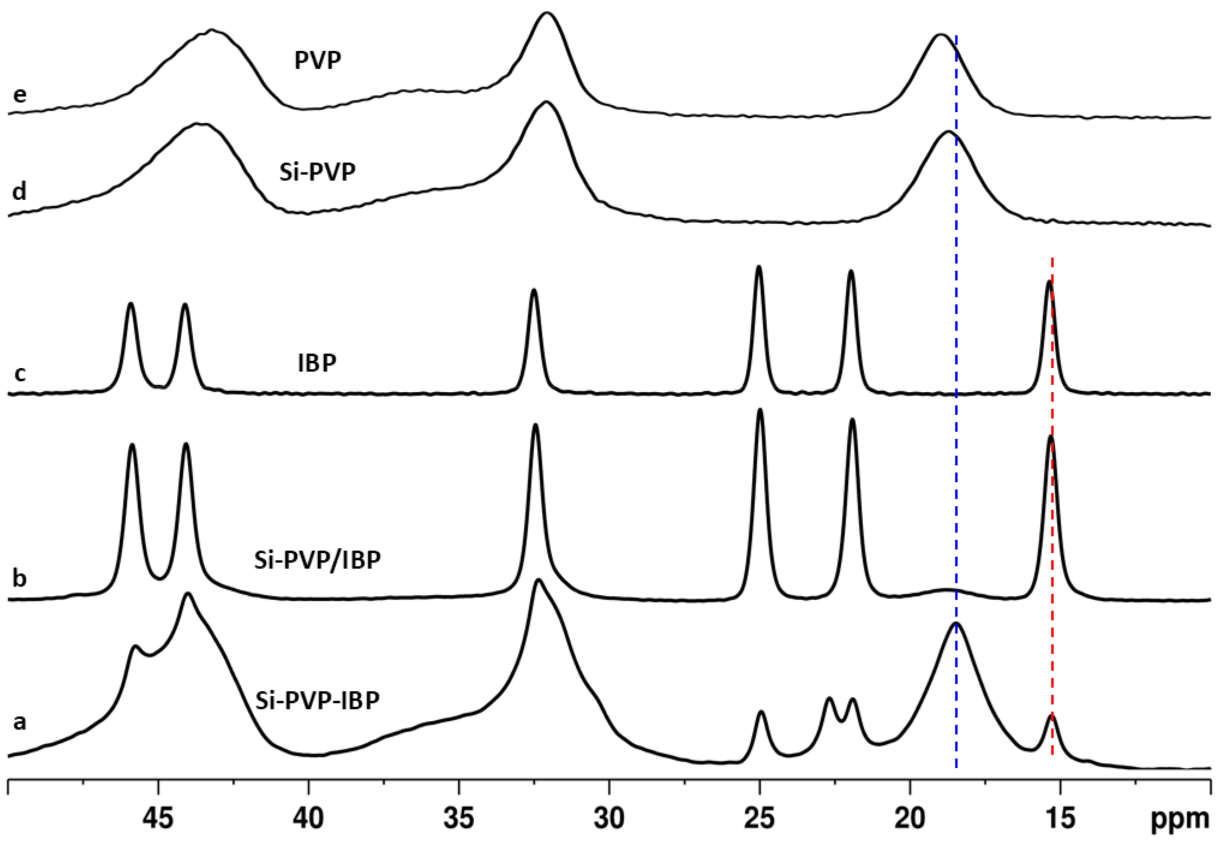

Figure S2. Expanded region of the aliphatic resonances in the $^{1}H \rightarrow ^{13}C$ CP-MAS spectra of: (**a**) Si-PVP-IBP hybrid, (**b**) Si-PVP/IBP adsorbate, (**c**) bulk IBP, (**d**) Si-PVP hybrid and (**e**) PVP. The dashed lines are guides for the eye for the chemical shift changes of selected resonances of IBP (red) and PVP (blue).
